# Transcriptomic data of MCF-7 breast cancer cells treated with G1, a G-protein coupled estrogen receptor (GPER) agonist^✩^

**DOI:** 10.1016/j.dib.2022.107948

**Published:** 2022-02-13

**Authors:** Uttariya Pal, Ankur Sahu, Pankaj Barah, Anil Mukund Limaye

**Affiliations:** a Department of Biosciences and Bioengineering, Indian Institute of Technology Guwahati, Guwahati - 781039, Assam, India; b Department of Molecular Biology and Biotechnology, Tezpur University, Tezpur 784028, Assam, India

**Keywords:** GPER, G1, RNA-seq, MCF-7

## Abstract

Besides short-term non-genomic effects, the G-protein coupled estrogen receptor (GPER) also mediates long-term genomic effects of estrogen. The genomic effects of GPER activation are not completely understood. G1 is a selective GPER agonist, which is popularly used for addressing the effects of GPER activation. Here, we present transcriptomic (RNA-seq) data on MCF-7 cells treated with 100 nM, or 1 µM G1 for a period of 48 h. The data are available from GEO (accession number GSE188706).

## Specifications Table

**Table:** 

Subject	Biological sciences
Specific subject area	Transcriptomics
Type of data	Table Transcriptomic data
How the data were acquired	RNA-seq
Data format	Raw sequence (FASTQ) Normalized count matrix
Parameters for data collection	MCF-7 breast cancer cells were treated with 100 nM G1 (n = 3), 1 µM G1 (n = 3), or vehicle (0.1% ethanol, n = 3) for 48 h.
Description of data collection	Total RNA was isolated. Sequencing libraries were prepared using standard protocol. Libraries were sequenced using the Illumina platform.
Data source location	Indian Institute of Technology Guwahati, Guwahati, Assam, India (26°11′14′′N 91°41′30′′E)
Data accessibility	Raw sequence, and processed data are submitted in Gene Expression Omnibus (GEO, accession number GSE188706). Direct URL to data: https://www.ncbi.nlm.nih.gov/geo/query/acc.cgi?acc=GSE188706

## Value of the Data

• This dataset captures the transcriptomic profiles of MCF-7 cells treated with vehicle, or the GPER agonist G1.
• It will contribute to a better understanding of the genomic effects of G1 in breast cancer cells.
• Investigators interested in GPER biology can use, and independently analyze, the data for insights into the genomic effects of GPER activation.

## 1 Data Description

Total RNA was isolated from MCF-7 cells treated with vehicle (0.1% ethanol), 100 nM G1, or 1 µM G1 for 48 h, and RNA-seq was performed. Quality assessment data for the RNA samples, including RNA concentration, RIN value, and the fragment length distribution, is provided as Supplementary data 1 and 2. The Raw FASTQ files are submitted in GEO with accession number GSE188706. HISAT2 was used to map the generated raw reads. Table 1 shows the summary of the read quality data including reads passed after filtering, reads failed due to low quality, reads failed due to too many Ns, reads with adapter trimmed using fastP, bases trimmed due to adapters using fastP, overall alignment rate using HISAT2 (in %).

**Table 1:** Summary of the sequencing read data.

Treatment	Sample	Reads passed after filtering	Reads failed due to low quality	Reads failed due to too many N	Reads with adapter trimmed using fastP	Bases trimmed due to adapters using fastP	Overall alignment rate using HISAT2 (in %)
**Vehicle (0.1% ethanol)**	**A1**	15151200	478998	5812	1606080	29796912	94.25
	**A2**	14885494	468992	5608	1329364	24873434	94.52
	**A3**	17318424	525390	6778	1711716	31229126	94.66

**100 nM G1**	**B1**	16088528	477180	6442	1664584	32398762	94.55
	**B2**	16267278	494750	6160	1601466	29507242	94.62
	**B3**	16615052	520318	6262	1703590	31487992	94.48

**1 µM G1**	**C1**	15197230	497706	5742	1105054	20382242	94.27
	**C2**	16917668	502368	6484	1603392	29159112	94.15
	**C4**	15618252	499354	6054	1468392	27198184	93.82

## 2 Experimental Design, Materials and Methods

### 2.1 Materials

Tissue culture flasks and dishes were purchased from Eppendorf (Hamburg, Germany). Dulbecco's Modified Eagle's Medium (DMEM) with phenol-red, and Dulbecco's Phosphate-Buffered Saline (DPBS) were from HiMedia (Mumbai, India). Fetal bovine serum (FBS) was from Gibco (NY, USA). 100X Pen-Strep antibiotic solution, and trypsin-EDTA were purchased from HiMedia (Mumbai, India). G1 was purchased from Cayman Chemical Company (Cat. #10008933, CAS# 881639-98-1, Michigan, USA). All other chemicals were purchased from SRL (Mumbai, India), or Merck (Mumbai, India).

### 2.2 Cell culture

MCF-7 cells were from NCCS (Pune, India). They were cultured in DMEM supplemented with 10% heat-inactivated FBS, 100 U/mL penicillin, and 100 µg/mL streptomycin (M1 medium) in a CO_2_ incubator maintained at 37°C, and 5% CO_2_.

### 2.3 Treatment

2 × 10^5^ MCF-7 cells were seeded in 35 mm dishes in M1 medium. After 48 h, the spent M1 medium was removed. The monolayer of cells was washed with DPBS, and treated with M1 medium containing 0.1% ethanol (vehicle control), 100 nM G1, or 1 µM G1. After 48 h of treatment, the cells were washed with DPBS before isolation of total RNA.

### 2.4 RNA samples

Cells were lysed in RNA extraction reagent prepared in-house. Lysates from two replicate dishes were pooled for purification of each RNA sample using the method described by Chomczynski and Sacchi [1] with modifications. Quality assessment data for the RNA samples is provided as Supplementary data 1 and 2. The RNA samples were then subjected to library preparation and sequencing on the Illumina plaform.

### 2.5 Library preparation

1 µg of total RNA was used to enrich mRNA using NEB Magnetic mRNA Isolation Kit (NEB, USA). Using fragmentation buffer, the enriched mRNA was fragmented (approximately 200 bp) and reverse transcribed into double-stranded cDNA using random primers. 1.8X Ampure beads (Beckman Coulter, USA) were used to purify the double-stranded cDNA fragments. The purified cDNA was ligated to the adaptor. The adaptor-ligated DNA was purified using Ampure beads, and enriched with specific primers compatible with Illumina platforms. The transcriptome library was constructed with the NEB Ultra II RNA Library Prep Kit (NEB, USA). The final enriched library was purified, quantified by Qubit (Thermo Fisher Scientific, USA), and anlayzed by 2100 Bioanalyzer (Agilent, USA). The library was sequenced with the Illumina NextSeq 500 paired end technology.

### 2.6 Reads quality check and trimming

The raw reads were in FASTQ format. The quality of the reads were assessed using fastqc tool [2]. The adapters, and low quality reads were filtered out from the FASTQ files using fastp tool [3]. Three criteria were set to filter out the low quality reads: removed reads lower than Q30 phred score; discarded reads shorter than 15 bp; Illumina adapter clipping. The fastqc tool was used to re-assess the filtered reads prior to mapping. The FASTQ files after the quality trimming and assessment were used for mapping. The number of read pairs passed after quality trimming and assessment are shown in Table 1.

### 2.7 Mapping reads against the human genome

The Ensemble *Homo sapiens* GRCh38 genome was used as reference genome for mapping the clipped reads (https://asia.ensembl.org/Homo_sapiens/Info/Index). Prior to mapping, indexing of reference genome was done using HISAT2 indexing scheme based on the Burrows-Wheeler transform and the Ferragina-Manzini (FM) index. Subsequently, clean reads were mapped using the HISAT2 tool against the index file [4]. The mapped output files (sam files) were converted into binary files (bam files) using Samtools [5].

### 2.8 Quantification of mapped reads

The Subread package with the featureCounts tool was used for quantification of mapped reads [6]. Mapped reads were counted at the feature (gene) level with the help of *Homo sapiens* GRCh38 annotation file (gtf).

## Ethics Statements

The work was carried out on a cell line, and did not involve animals or human subjects.

## CRediT authorship contribution statement

**Uttariya Pal:** Data curation, Methodology, Writing – review & editing. **Ankur Sahu:** Formal analysis. **Pankaj Barah:** Supervision, Formal analysis. **Anil Mukund Limaye:** Conceptualization, Supervision, Writing – review & editing, Funding acquisition.

## Declaration of Competing Interest

The authors declare that they have no known competing financial interests or personal relationships that could have appeared to influence the work reported in this paper.

## Appendix Supplementary materials

Image, application 1

Image, application 2
