# Supplementary material for: Transcriptomic data of MCF-7 breast cancer cells treated with G1, a G-protein coupled estrogen receptor (GPER) agonist
Source: Data Brief. 2022 Feb 13;41:107948. doi: 10.1016/j.dib.2022.107948 (PMC8866881; doi:10.1016/j.dib.2022.107948)
Supplement: Supplementary file 1 [file mmc1.pdf]

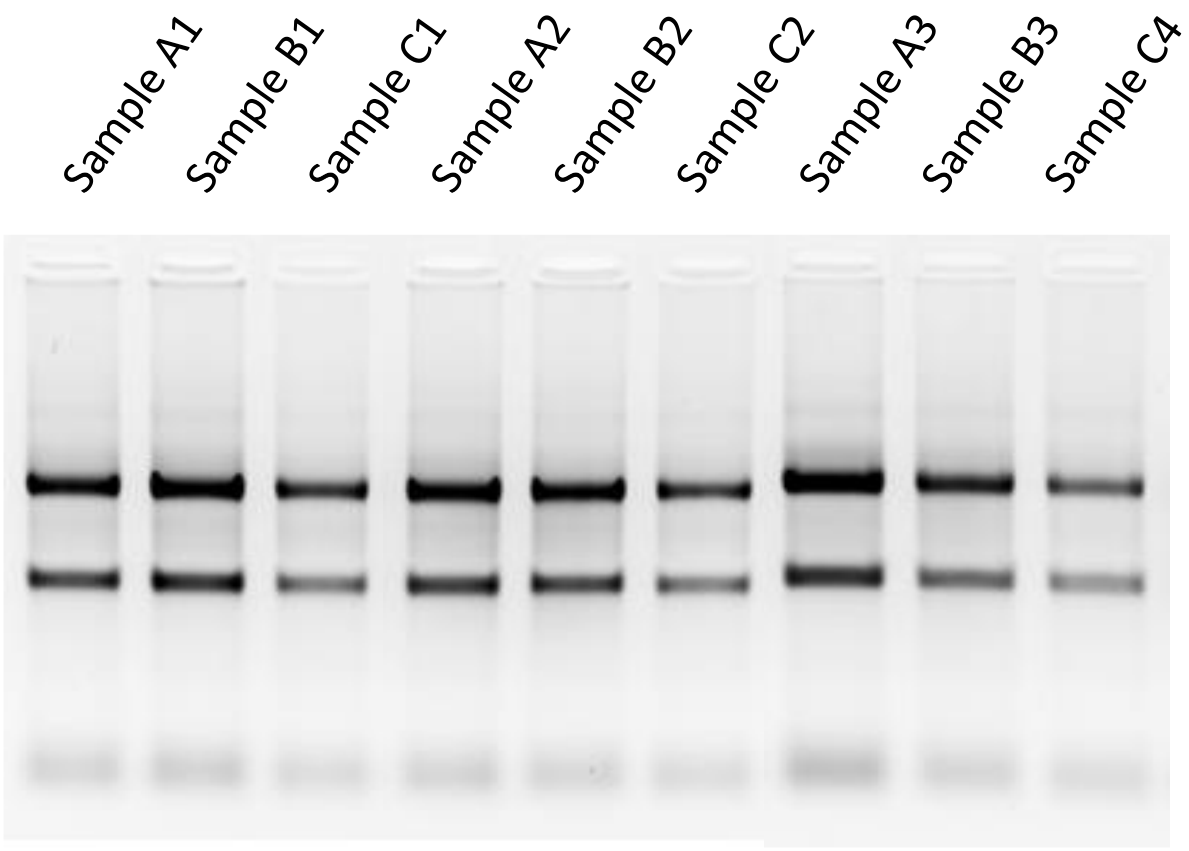

Supplementary Data 1: Images of total RNA before library preparation and sequencing from MCF-7 cells treated with with 0.1% ethanol (samples A1, A2, and A3), 100 nM G1 (samples B1, B2, and B3), or 1 µM G1 (samples C1, C2, and C4) for 48 h.
