# Supplementary material for: Transcriptomic data of MCF-7 breast cancer cells treated with G1, a G-protein coupled estrogen receptor (GPER) agonist
Source: Data Brief. 2022 Feb 13;41:107948. doi: 10.1016/j.dib.2022.107948 (PMC8866881; doi:10.1016/j.dib.2022.107948)
Supplement: Supplementary file 2 [file mmc2.pdf]

EL1: Electronic Ladder

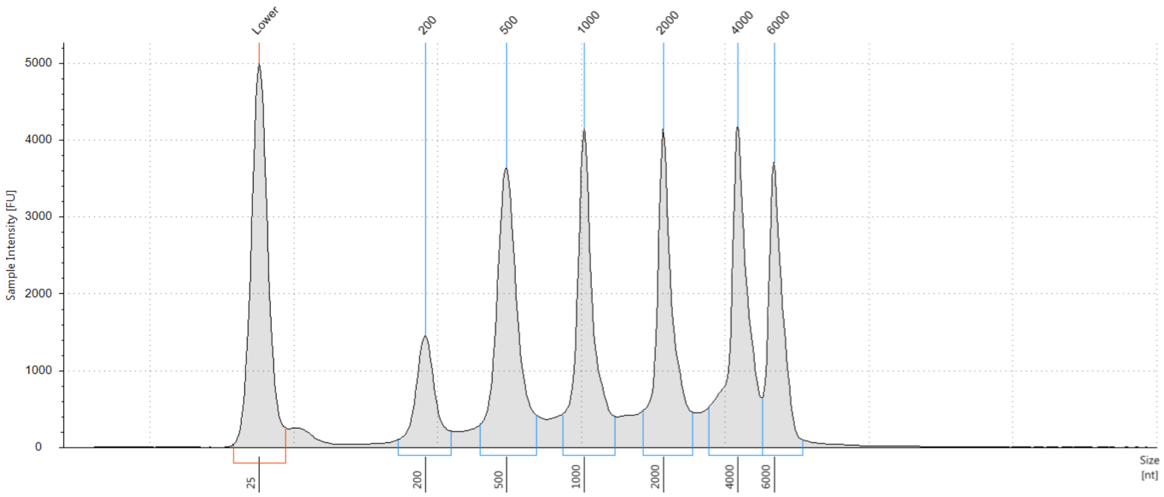

Sample Table

RINe	Sample Description
-	Electronic Ladder

Peak Table

Size [nt]	Calibrated Conc. [ng/µl]	Assigned Conc. [ng/µl]	Peak Molarity [nmol/l]	% Integrated Area	Peak Comment
25	40.0	40.0	4710	-	
200	6.82	-	100	7.80	
500	18.3	-	107	20.88	
1000	16.3	-	47.9	18.62	
2000	15.8	-	23.3	18.11	
4000	17.8	-	13.1	20.38	
6000	12.4	-	6.10	14.22	

 © Agilent Technologies, Inc. 2016

Sample A1

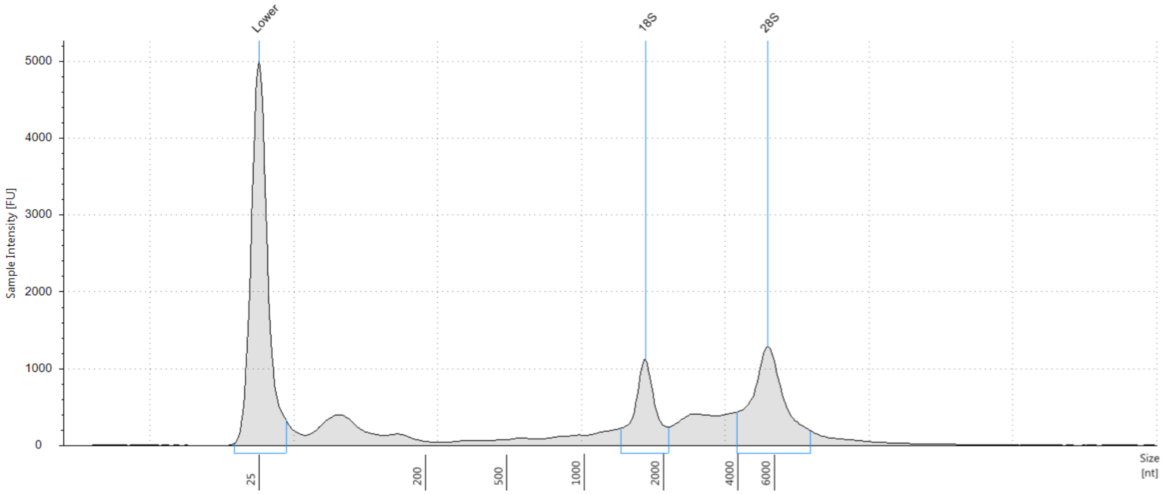

Sample Table

RINe	Sample Description
8.5	Sample_A1

Peak Table

Size [nt]	Calibrated Conc. [ng/µl]	Assigned Conc. [ng/µl]	Peak Molarity [nmol/l]	% Integrated Area	Peak Comment
25	40.0	40.0	4710	-	
1713	5.94	-	10.2	34.92	
5604	11.1	-	5.81	65.08	

 © Agilent Technologies, Inc. 2016

Sample A2

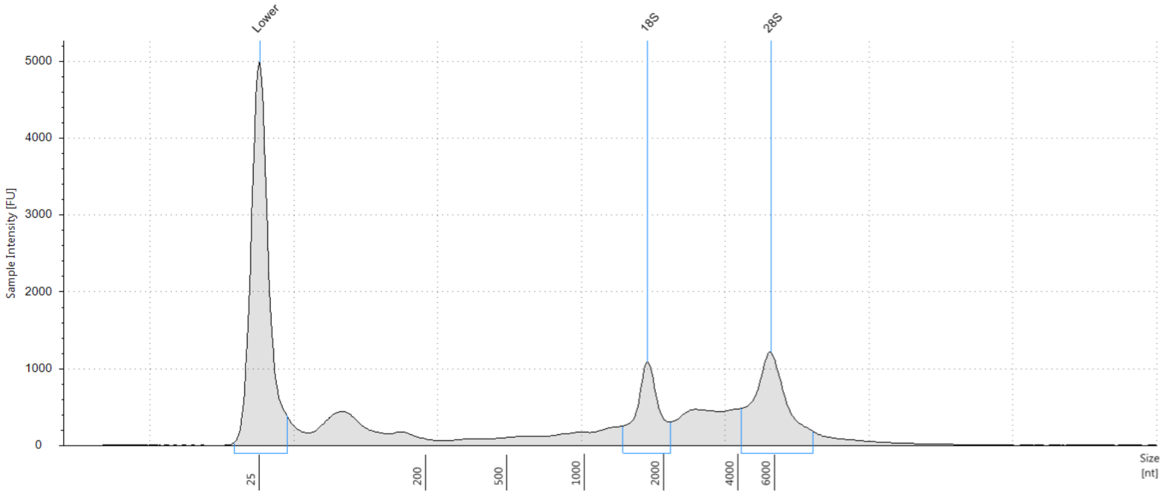

Sample Table

RINe	Sample Description
8.0	Sample_A2

Peak Table

Size [nt]	Calibrated Conc. [ng/µl]	Assigned Conc. [ng/µl]	Peak Molarity [nmol/l]	% Integrated Area	Peak Comment
25	40.0	40.0	4710	-	
1741	5.82	-	9.84	36.95	
5783	9.93	-	5.05	63.05	

 © Agilent Technologies, Inc. 2016

Sample A3

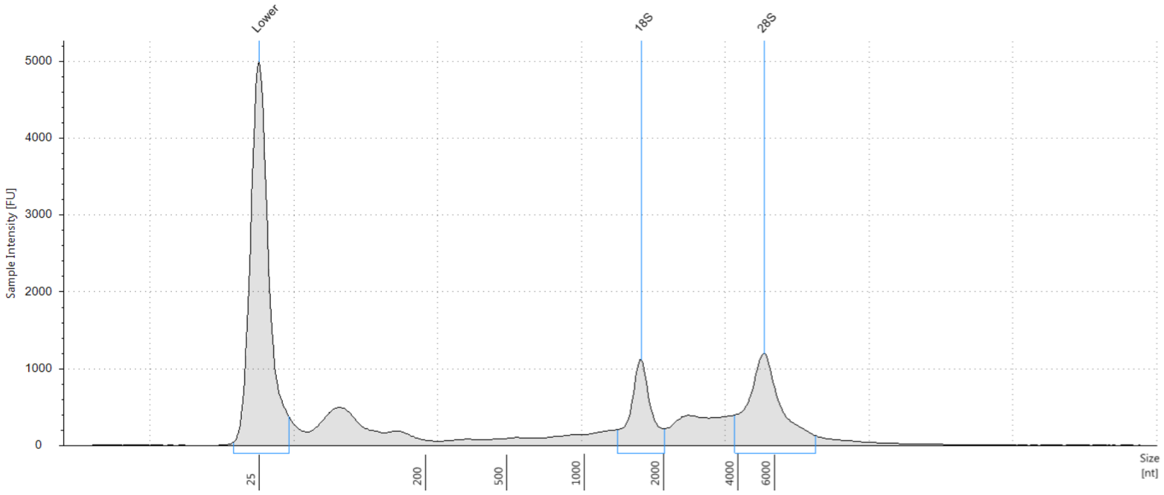

Sample Table

RINe	Sample Description
8.5	Sample_A3

Peak Table

Size [nt]	Calibrated Conc. [ng/µl]	Assigned Conc. [ng/µl]	Peak Molarity [nmol/l]	% Integrated Area	Peak Comment
25	40.0	40.0	4710	-	
1655	5.15	-	9.16	35.69	
5420	9.28	-	5.04	64.31	

 © Agilent Technologies, Inc. 2016

Sample B1

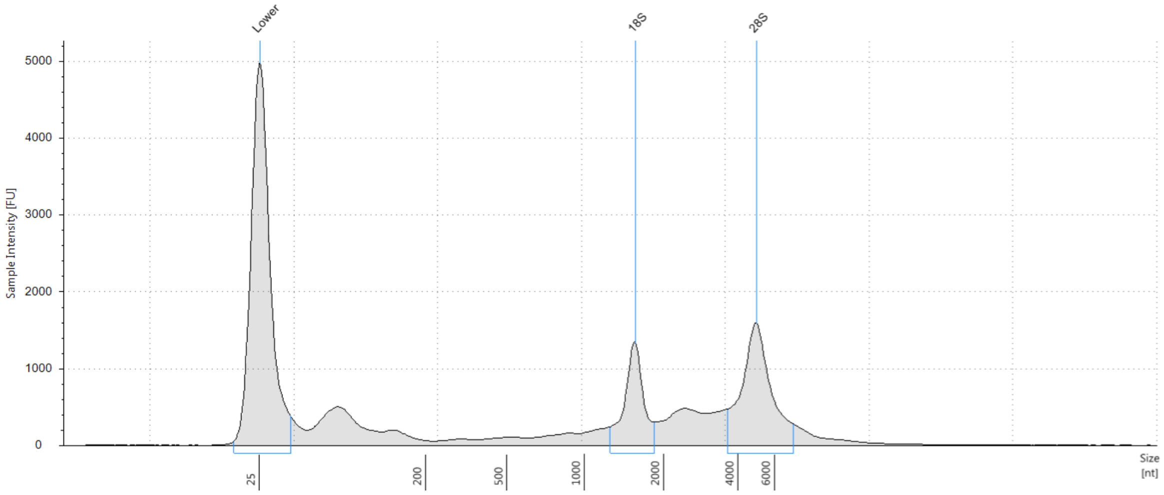

Sample Table

RINe	Sample Description
8.7	Sample_B1

Peak Table

Size [nt]	Calibrated Conc. [ng/µl]	Assigned Conc. [ng/µl]	Peak Molarity [nmol/l]	% Integrated Area	Peak Comment
25	40.0	40.0	4710	-	
1558	5.69	-	10.7	34.82	
4906	10.6	-	6.38	65.18	

 © Agilent Technologies, Inc. 2016

Sample B2

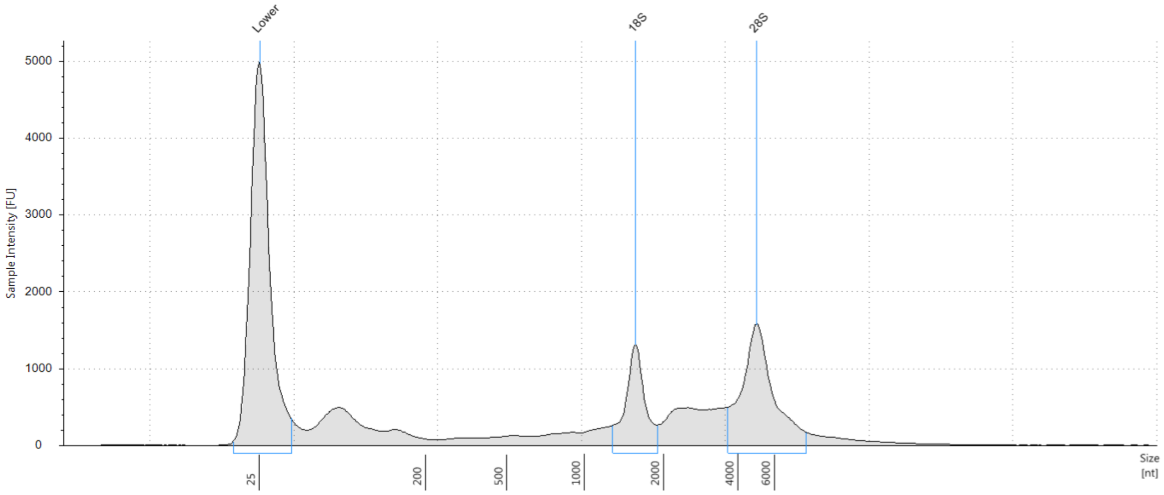

Sample Table

RINe	Sample Description
8.5	Sample_B2

Peak Table

Size [nt]	Calibrated Conc. [ng/µl]	Assigned Conc. [ng/µl]	Peak Molarity [nmol/l]	% Integrated Area	Peak Comment
25	40.0	40.0	4710	-	
1561	5.54	-	10.4	33.42	
4928	11.0	-	6.58	66.58	

 © Agilent Technologies, Inc. 2016

Sample B3

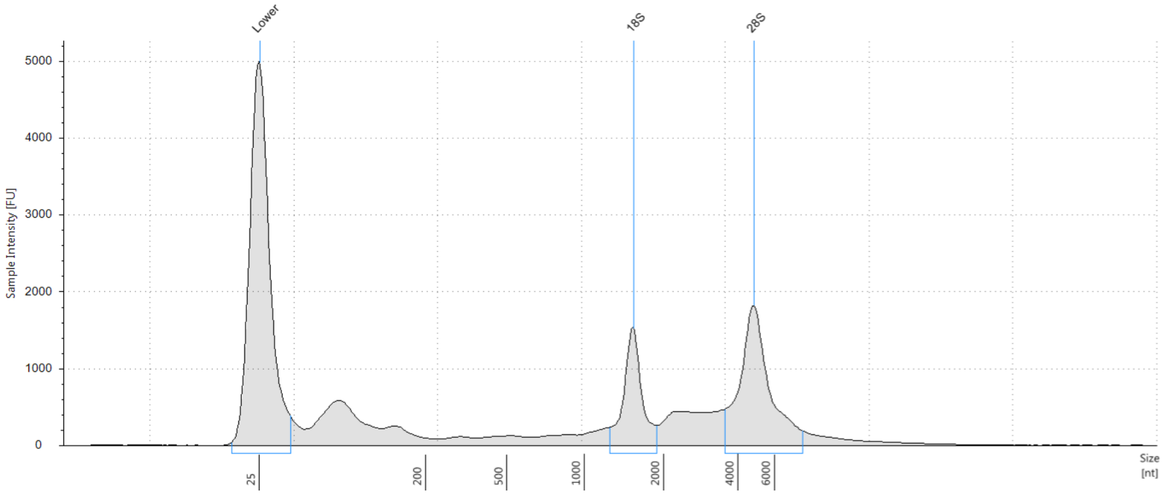

Sample Table

RINe	Sample Description
9.1	Sample_B3

Peak Table

Size [nt]	Calibrated Conc. [ng/µl]	Assigned Conc. [ng/µl]	Peak Molarity [nmol/l]	% Integrated Area	Peak Comment
25	40.0	40.0	4710	-	
1540	5.91	-	11.3	33.91	
4796	11.5	-	7.07	66.09	

 © Agilent Technologies, Inc. 2016

Sample C1

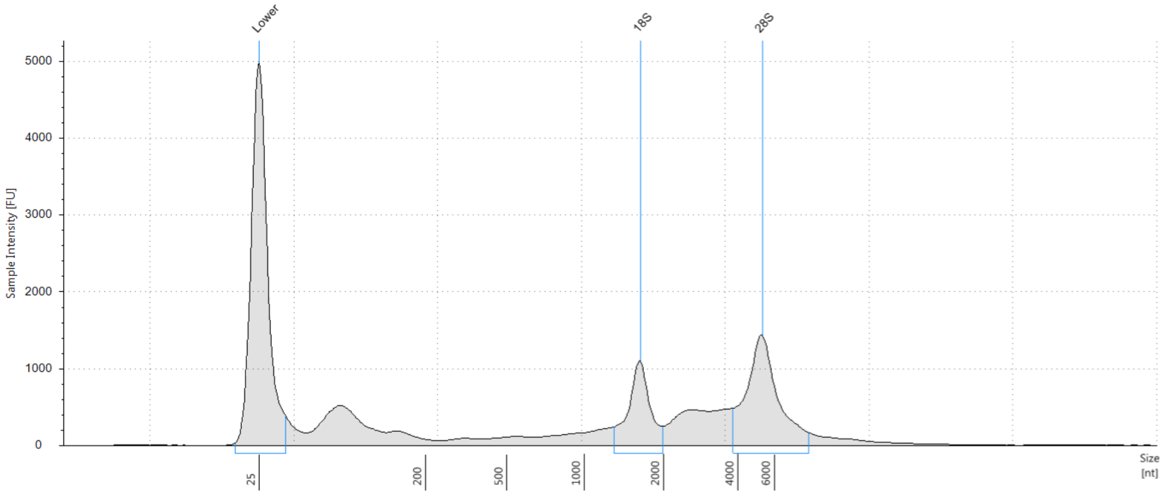

Sample Table

RINe	Sample Description
8.2	Sample_C1

Peak Table

Size [nt]	Calibrated Conc. [ng/µl]	Assigned Conc. [ng/µl]	Peak Molarity [nmol/l]	% Integrated Area	Peak Comment
25	40.0	40.0	4710	-	
1635	6.18	-	11.1	34.01	
5258	12.0	-	6.71	65.99	

 © Agilent Technologies, Inc. 2016

Sample C2

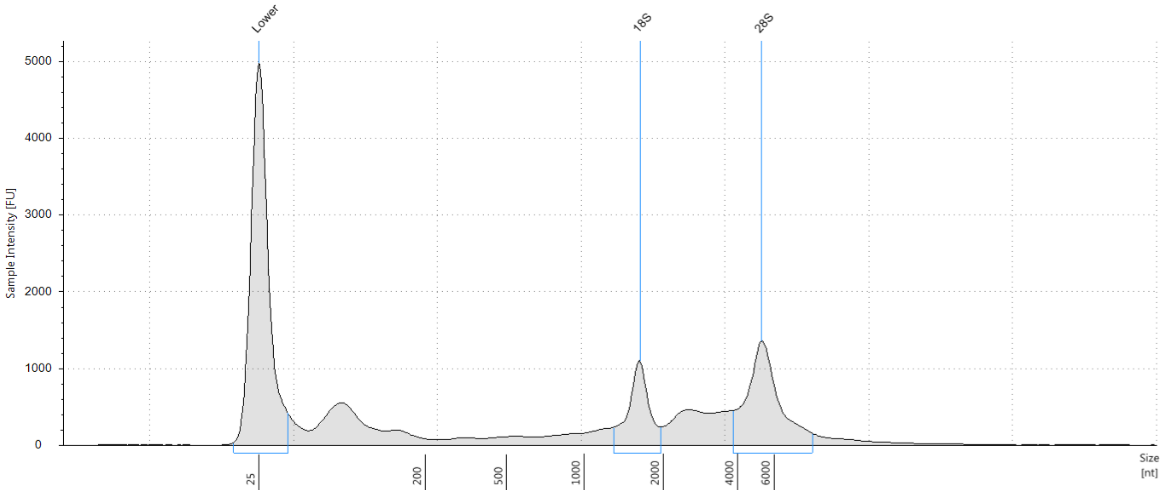

Sample Table

RINe	Sample Description
8.2	Sample_C2

Peak Table

Size [nt]	Calibrated Conc. [ng/µl]	Assigned Conc. [ng/µl]	Peak Molarity [nmol/l]	% Integrated Area	Peak Comment
25	40.0	40.0	4710	-	
1632	5.50	-	9.91	33.97	
5239	10.7	-	6.00	66.03	

 © Agilent Technologies, Inc. 2016

Sample_C4

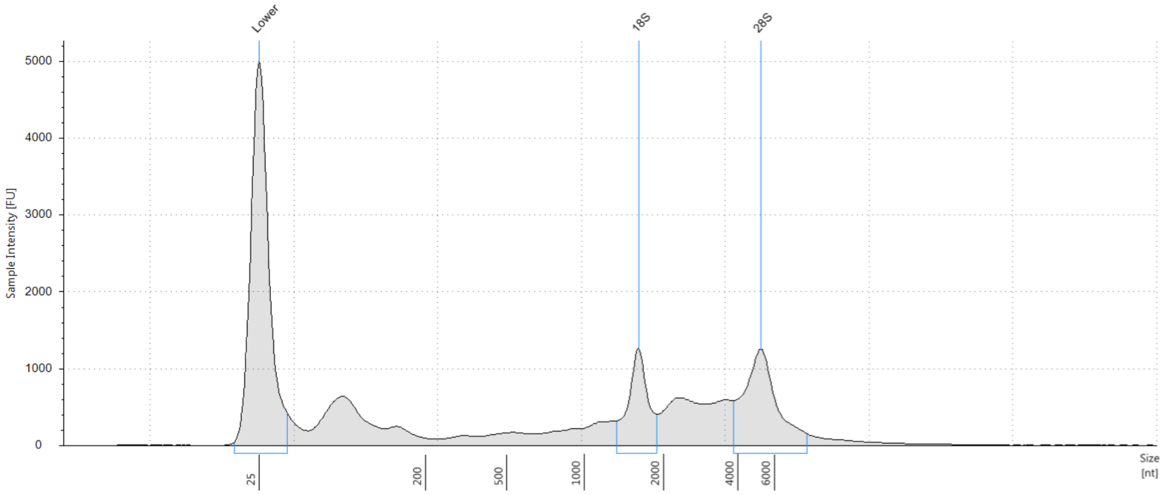

Sample Table

RINe	Sample Description
7.7	Sample_C4

Peak Table

Size [nt]	Calibrated Conc. [ng/µl]	Assigned Conc. [ng/µl]	Peak Molarity [nmol/l]	% Integrated Area	Peak Comment
25	40.0	40.0	4710	-	
1616	5.79	-	10.5	37.12	
5172	9.81	-	5.58	62.88	

 © Agilent Technologies, Inc. 2016
